# Oral Health and Its Aetiological Role in Behçet's Disease

**DOI:** 10.3389/fmed.2021.613419

**Published:** 2021-05-20

**Authors:** Gonca Mumcu, Farida Fortune

**Affiliations:** ^1^Department of Health Management, Faculty of Health Science, Marmara University, Istanbul, Turkey; ^2^Centre for Immuno-Biology and Regenerative Medicine, Behçet's Centre of Excellence, Barts and the London School of Medicine and Dentistry, Queen Mary University of London, London, United Kingdom

**Keywords:** oral health, aetiology, oral ulcer, immune response, Behçet's disease

## Abstract

Behçet's disease (BD) is a chronic multi-systemic inflammatory disorder characterised by oro-genital ulcers, cutaneous manifestations, ocular, vascular, neurologic and gastrointestinal involvement. Complex interactions operating on the genetic background e.g.(HLA51), of infectious and other environmental agents, together with immune dysregulation impacts on the pathogenesis of BD. This suggests that the environmental factors triggering immune responses may activate clinical manifestations in genetically susceptible individuals. Since oral health forms the basis of all general health both dental and systemic, it is an important component of both Dentistry and Medicine. Oral ulcers are the most common clinical manifestation of oral mucosal health. Changes in the oral environment consequently acts as an infective and immune trigger. In this review, complex interactions between the oral ulcers, the oral microbiome and immune responses together with the course of oral and systemic disease manifestations in BD are discussed in the context of the aetiologic role of oral health.

## Introduction

Behçet's disease (BD) is a chronic multi-systemic inflammatory disorder characterised by mucocutaneous manifestations and involvement of musculoskeletal, vascular, ocular, central nervous and gastrointestinal systems (1–5). Oral ulcer is the most common and usually initial symptom of the disease. Recurrent exacerbations as well as remissions with unpredictable clinical manifestations are the hallmark of BD. Genetic susceptibility (HLA-B51), infectious agents (bacteria and viruses e.g., *Streptococcus* spp., Herpes simplex virus), hormones and immune dysregulation are implicated in the aetiopathogenesis of BD (1–4). Infections may play a pivotal role in the initiation of the disease and relapses of clinical manifestations in genetically susceptible patients (6–13).

The higher prevalence of BD is present in Mediterranean and Middle East countries along with Ancient Silk Route. Environmental factors tend to be specific to geographic areas and reflect the interactions between genetic factors and infective organism with host factors in BD (4, 14, 15). Both epidemiologic and hospital-based studies were carried out to investigate the role of environmental factors in Turkish, African and Asian immigrants. The results show that the prevalence of BD is higher in immigrants compared to individuals living in the same country, while it is lower than that of their country origin (16–18). Furthermore, there has been a decrease in the prevalence of BD, with a milder clinical course in both the Japanese and Korean populations who have minimal ethnic diversity (19–23). These factors may be important pointers to understanding the impact of environmental factors in BD (15–26).

## Oral Environment

Oral health is the bridge between Dentistry and Medicine, and consequently closely related to systemic health. The oral environment provides a dynamic ecosystem which includes mucosal surfaces, saliva, local and systemic immune system and species of bacteria, viruses and fungi, and the resulting complex interactions. The oral microorganisms are found on mucosal surfaces, saliva and biofilm around the tooth surface. This results in the oral mucosa being constantly exposed to antigenic stimulation with commensal and/or pathogenic oral microbial antigens (27). Comorbidities, diet and life style, ethnicity, medication including immune suppressive drugs, broad-spectrum antibiotics used for ulcers, polypharmacy affecting the salivary flow rate and oral health status (natural teeth vs. edentulous) all collectively may affect the microbiome ecosystem within the mouth (5, 24, 27–37).

**Figure 1:**
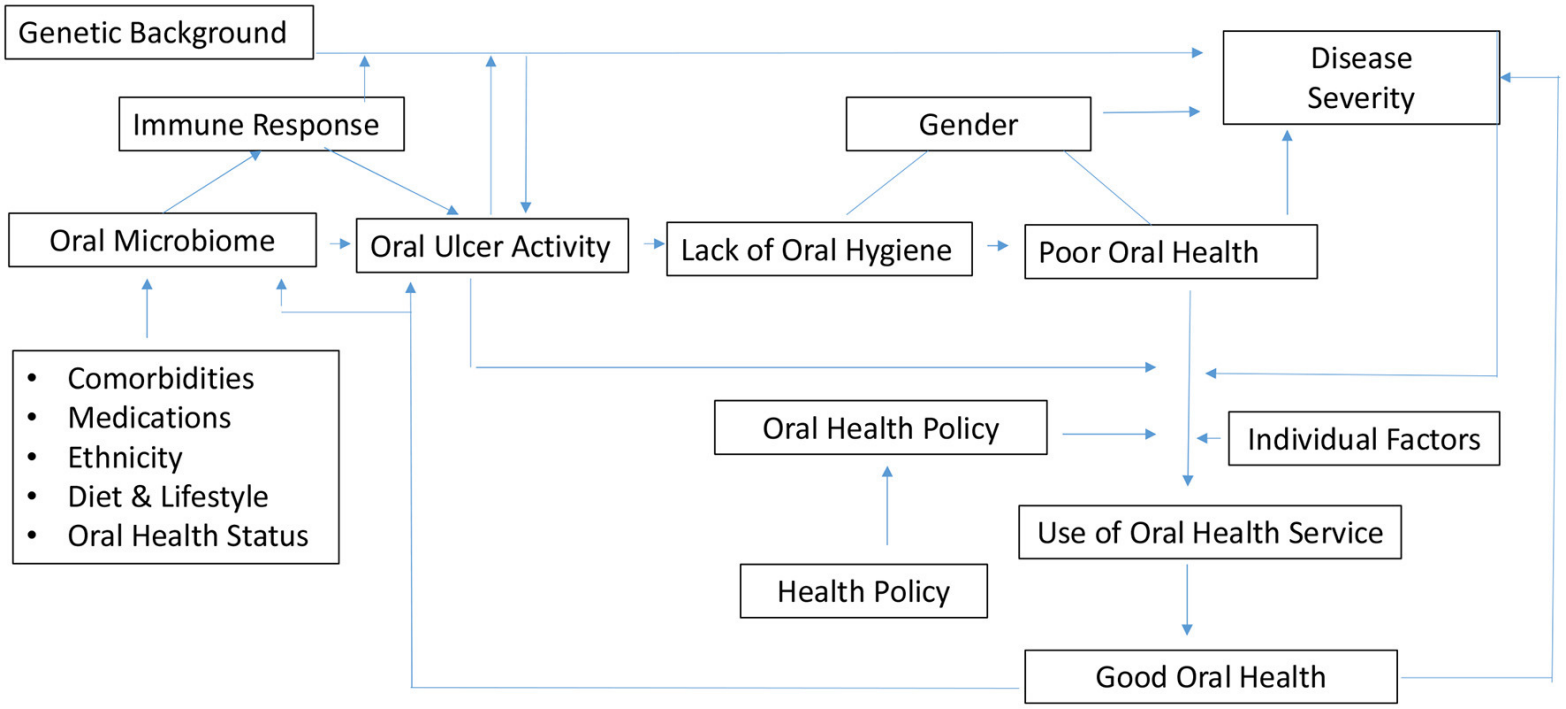
Overview of oral health as part of the aetiopathogenesis in Behçet's disease.

Microorganisms play a critical role in maintaining the balance of health and disease as commensal microorganisms protect the mucosal barrier and prevent pathogenic colonisation (38–42). Although the oral mucosa is an effective physical barrier for microorganisms and their antigens (27), molecular mimicry and cross-reactivity between tissue antigens, microbial antigens and immune dysregulation can trigger oral ulcer activity in BD (10, 11, 27, 43). Moreover, genetic factors also have a role in the occurrence of oral ulceration as shown by the overlapping genetic risk factors in patients with BD and Recurrent Aphthous Stomatitis (RAS) in genome wide association studies (44, 45) (Figure 1).

## Oral Microbiome

Although Oral microorganisms can trigger the immune system and inflammatory pathways, they contribute to the aetiopathogenesis of BD. Infective oral foci caused by oro-dental and periodontal disease are also part of the environmental factors affecting the pathogenesis of BD (29, 31, 46, 47) (Figure 1). Although some *streptococcal* strains are the main focus in the aetiology of BD, other organisms including *herpes simplex*, human *cytomegalovirus, Epstein-Barr*, and *varicella zoster* viruses are all thought to be aetiologic factors in BD and contributes to the induction of oral ulceration in BD (15, 24–26, 28, 30, 41, 42, 48, 49).

The relationships between *Streptococcal* infections and clinical observations may be summarised by high prevalence of tonsillitis and dental caries, and the activation of symptoms by dental treatment and bacterial plaque (6–11, 50, 51). Increases in the colonisations of *Streptococci sanguis* (*S. sanguis*) on tongue, dental plaque and buccal mucosa as well as *S. mitis* and *S. salivarius* within the dental plaque and saliva are observed in BD (6, 11, 13, 52). In addition, there is a beneficial effect of antimicrobial treatment on both mucocutaneous and systemic manifestations (50, 53, 54).

Salivary *S. mutans* colonisation is the main aetiological factor for dental caries and is thought to be important pathogen with increased colonisation in active oral ulceration in BD (55). Salivary *S. mutans* levels are found to be high in male patients who have a more severe disease course (55), have worse periodontal indices (29). They also have decreased levels of serum mannose binding lectin (MBL <100 ng/ml) a host defence protein with raised levels of *S. mutans* colonisation in saliva (55). The low serum MBL associated with oral infections and oral ulcer activity, is an indication that there is dysregulation of innate immunity (55) and severe disease course (56).

There is an increase in *S. sanguis* colonisation in oral environment with uncommon serotypes which cross-react with epithelial cells (11). The adhesion of *S. sanguis* to buccal epithelial cells is a critical activating factor in BD (2, 11, 12, 43). Oral and ocular inflammation are also induced by *S. sanguis* infection, after heat trauma to the oral mucosa in a germ-free mice model (6). In BD, stimulation of peripheral blood T cells using KTH-1 (a crude extract of *S. sanguis*) results in an increase of interleukin-6 (IL-6) and interferon-gamma (IFN-γ) (57). In addition, stimulated KTH-1 γδ T-cell lines secrete IL-8 and tumour-necrosis factor-alpha (TNF-α) (58). These infective agents use the sophisticated mechanism of molecular mimicry, the microbial heat shock proteins (*S. sanguis HSP-65*) shares homology with human HSP-60 protein to stimulate human immune system and influence the disease pathogenesis of BD (59, 60).

## Innate Immunity

Neutrophils as the first line defender against infections have important roles in the innate immunity of BD. The ulcers initiate recruitment of neutrophils to the site of increased activity. Chemotaxis and phagocytosis, as well as superoxide production are increased in hyperactive neutrophils in BD. Elevated neutrophil extracellular traps (NETs) production caused by neutrophil activation are seen in disease activity and contributes to vasculitis and thrombosis (61). Damaged tissues also act as triggering agents and contribute to immune responses (14). Neutrophil elastase (NE) is enzymatically active in saliva and responsible for the majority of protease activity. The combination of Colchicine and Azathioprine which are effective medications for the treatment of oral ulcers help the regulation of NE activity for oral ulcer in Behçet's patients (34). Serum S100 calcium-binding protein A12 (S100A12) constitutively expressed on neutrophils and secreted when neutrophils are activated has inflammatory functions with potent chemotactic activity and contributes to the BD pathogenesis (62).

It is well-known that Toll-like receptors (TLRs) and pathogen-recognition receptors (PRRs) play critical roles in the pathogenesis of inflammatory diseases. Lipoteichoic acid (LTA) from the cell wall of gram-positive bacteria is recognised by TLR and PRRs. The oral microbial community is recognised as a potential trigger of TLR2 and TLR4 in BD (63). BD patients with *S. sanguis* and *S. feacalis* have high serum levels of IgG anti-LTA antibody. In addition, LTA stimulates IL-8 production in peripheral blood mononuclear cells (64).

Saliva includes antibacterial, antiviral and antifungal properties which arise from its variety of constituent proteins which include lysozyme, lactoferrin, lactoperoxidase, statherin, immunoglobulin A and mucin (27). Antimicrobial peptides in saliva originates from the neutrophils and epithelial cells. Salivary human neutrophil defensin HNP- 1–3 and S100 molecules are increased in BD patients with a more severe disease (65, 66), and salivary HNP 1–3 levels are associated with oral ulcer activity (66). The antimicrobial cathelicidin, LL37, is produced from mucosal epithelial cells and found in saliva is associated with the number of oral ulcers (65). While an increase in the levels of vascular endothelial growth factor (VGEF) is observed in active oral ulcers, unlike epithelial growth factor and transforming growth factor-alpha levels which are found to be low (67). An increase in salivary IL-1α levels is seen in BD compared to healthy controls, as well as elevated serum levels of salivary IL-6 levels (68) (Figure 1).

## Adaptive Immune Responses

Microorganisms accumulate at the mucosal surfaces and penetrate the epithelial barrier. The intraepithelial γδ cells usually protects the mucosal barrier by producing antimicrobial peptides and activating mucosal immune responses (69, 70). Although most of the work on γδ T cells relate to Vgamma9 and Vdelta2 chains, other γδ T cells subsets which are activated by microbial products (69) as pathogenic strains of *S. sanguis* and *S. mitis*, are probably more important because they mainly populate in the oral environment (10). Once the pathogenic microorganisms are eliminated by neutrophils the consequent bacterial products are recognised by γδ T cells that stimulate pro-inflammatory cytokines from monocytes (15, 69). After innate immune responses are activated by pathogens, vasculitis and tissue damage results from neutrophil T cells activation BD (39). The Th17 axis influences the cross talk between lymphocytes and neutrophils in BD (71). The expression of cytokeratin and adhesion molecules are upregulated in oral ulcers in BD resulting in interaction between cell-cell and the cell-extracellular matrix (72) (Figure 1).

Immunoglobulin G (IgG) responses to *Actinomyces viscosus, S. mutans, S. sanguis, S. oralis and Eikenella corrodens, Campylobacter rectus and Prevotella intermedia* are found to be low in BD patients compared to those of healthy controls (73). As related with these results, poor periodontal health is related to decreases in antibody responses in BD (73). Moreover, IL-1 is implicated in the pathogenesis of both periodontitis and BD. In single nucleotide polymorphisms, IL-1alpha-889C allele levels are higher in BD than healthy controls (46). In addition, poor periodontal health is associated with age, male gender and carrying IL-1beta-511T allele in the Turkish population (46). One may speculate that BD severity is affected by the periodontitis-induced inflammatory response due to an alteration in the IL-1 gene (46). Similarly, TNF-alpha-1031T/C (CC genotype) polymorphism is higher in BD than healthy controls. Disease severity is associated to poor periodontal health and the TNF-alpha-1031T/C (CC genotype) polymorphism in BD (47) (Figure 1).

## Salivary Microbiome

Microbiome related studies indicates that the gut, oral or skin pathogens and their relationship with specific microbial communities affect with disease activity. They may act as both an aetiological trigger and a prognostic indicator in the disease pathogenesis. This occurs when dysbiosis of the established microbial community results in inflammation and a decrease in host pathogen resistance. Numerous microbiome studies have established the aetiopathogenesis of diseases (36, 38, 39, 74–76).

Immune response can be activated by changes in the established microbiome communities at the mucosal surface and their surface secretions in BD (e.g., salivary and gut mucous). Sequencing the microbiome of the saliva and gut have provided information about real-time monitoring of patients with disease-related markers. However, the geographic area, genetic/ethnic factors, lifestyle, diet pattern, dentition and oral health are all associated with the composition and biodiversity of the host's microbiota. The biodiversity pattern and the role of symbiosis in the course of the disease needs to be evaluated to understand the possible mechanisms in the pathogenesis of BD (38–42, 77–79) (Figure 1).

Our saliva microbiome study shows that ulcer sites of BD are more highly colonised with *S. salivarious* compared to those of RAS (42). However, increase in the colonisation of *Rothia dentocariosa* is found in the non-ulcer area of BD and RAS. Moreover, *S. salivarius* and *S. sanguis* heavily colonises on active oral ulcers in BD. In contrast, there is an increase in the colonisation of *Neisseria* and *Veillonella* are found in healthy controls (42). Our another study found an increase in *Haemophilus parainfluenzae* and decrease in *Alloprevotella rava. Leptotrichia* are also observed in salivary microbiome in BD. Interestingly, after dental and periodontal treatment, the salivary microbiome pattern stabilises to a more symbiotic pattern in short-term (41). Elevated abundances of *Dethiosulfovibrionaceae* and *Spirochaetaceae* families, with *Trepenema* TG5 at genus level being found in the oral microbiome from BD patient (38). Changes in microbiome profile modifying immune responses contribute to immune dysregulation during the development and progression of the diseases (38, 41, 42). These studies of the salivary microbiome provide information which may assist in the development of new therapies for use in BD (15, 38, 40–42) (Figure 1).

Similarly, dysbiosis of the gut microbiota activates the inflammatory response in BD (38, 39, 80, 81). Changes in the gut microbiota production decreases butyrate production. Butyrate importantly maintains the commensal IgA mucosal barrier preventing entry of pathogenic bacteria across the epithelial surface. In BD, it promotes Regulatory T cell (Treg) responses indicating a pivotal role in the anti-inflammatory response (39). In addition, faecal microbiota transplants in a mouse model causes the activation of, autoimmune uveitis, with an increase in the inflammatory cytokine responses such as IL-17 and IFN-γ (40).

## Oral Health and Disease Course

When the oral health of BD patients is evaluated, poor periodontal scores are observed, especially in males with the more severe course of the disease. An increase in the microbial plaque accumulation around teeth, gingival inflammation, periodontal tissue destruction and tooth loss are found in BD patients, compared to healthy controls. Using Regression analysis, the elevated microbial plaque accumulation is associated with oral ulcer activity and male gender. Moreover, an increase in plaque accumulation is found to be risk factor for a more severe disease course (29). Recently, these results are confirmed in a 5-years follow-up study using a Mediation analysis. The increase in the disease severity score which reflects organ involvement globally, is directly associated with male gender (31). Tooth extraction may be necessary as a last resort in the (male) patients with severe dental and advanced periodontal disease not amenable to treatment, as they act as mediators for elevated disease severity in BD (31) (Figure 1).

Oral health status varies between countries. The utilisation of oral and dental health services for patients is affected by both individual factors and local or national policies which may be part of the overall country specific National Health Policies (82). In our previous study, the number of monthly oral ulcers, filled teeth, frequency of tooth brushing and dental visits are found to be high in patients from UK compared with those in patients from Turkey (83). In addition, a lower utilisation rate of Dental Services and lack of oral hygiene lead to a poorer oral health in patients from Turkey (83) (Figure 1).

The elimination of oral infection foci by dental and periodontal treatment is an unacknowledged option for control of both oral ulcer activity and systemic manifestations (24, 26, 28, 30, 32, 46, 47). Oral ulcer activity is recorded pre-treatment period during a month. In the intervention group, the dental and periodontal treatments are carried out in 1-month period. In addition, control group is followed during study period. Oral hygiene education is given both groups and oral health is evaluated by indices. However, systemic treatments are not changed during the study period in both groups. Oral ulcer activity is initially increased in the treatment period owing to mechanical trauma and activation of systemic immune response, especially in the first 2 days. In contrast, the number of recurrent oral ulcers is significantly decreased. At 6 months, there is improvement of oral health in the intervention group when compared to the pre-treatment and treatment periods. Moreover, the number of recurrent ulcers is also lower than in the control group. Although a flare of oral ulcers is seen early, the overall the long-term outcome demonstrates that better oral hygiene is associated with decreased disease activity (32) (Figure 1). It should be borne in mind that traumatic dental and/or prosthodontic restorations including implants may also trigger oral ulcers with consequent activation of BD (27, 28, 32).

## Oral Ulcer and Treatment Protocols

Oral ulcers are a common clinical manifestation of BD. In addition, oral ulcer activity may persist during the disease process. When this occurs, the mucosal barrier is breached. This together with dysbiosis results in microorganisms and inflammatory mediators being released into the general circulation, and actively contribute to the ongoing activity of the disease process (15, 46, 47, 84) (Figure 1).

Regular oral hygiene and topical treatments are the preferred approach to reduce dysbiosis and stabilise the oral environment, to encourage ulcer healing and reduce disease activity. New BD mouthwash (Triorasol) has a positive effect on the oral ulcer pattern and quality of life during the use of 6 months. The mouthwash reduces pain and clears the ulcers within 10–14 days and should be continued in short term until cropping of ulcers ceases. An additional benefit is that it reduces oral and oropharyngeal scarring commonly seen in BD. The efficacy of this mouthwash is found to be high compared to betamethasone mouthwash in BD (85). In addition, immunosuppressive medications for the elimination of oral ulcers in resistant mucocutaneous cases can improve outcomes in a multidisciplinary setting (5, 84, 86–88).

Antibiotics also have a place in ulcer control of mucocutaneous lesions especially when there is an infectious aetiology, especially streptococcal infections. Minocycline has modest effect on oral ulcer activity. In addition, IL-1β and IL-6 responses from peripheral blood mononuclear cells (PBMC) stimulated with streptococcal antigen, are decreased by using this antibiotic (50). In another study, colchicine combined with prophylactic benzathine penicillin is found to be effective in the reducing the frequency and duration of oral ulceration compared to those of colchicine alone (89).

Macrolides are another therapeutic option because of its immunoregulatory and antimicrobial effects. They accumulate intracellularly and are transferred to the inflammatory area by neutrophils. An example of this, Azithromycin which decreased the healing time (53) and the number of oral ulcers (54). Good oral hygiene also shortened the healing time and the microbial plaque accumulation around teeth. Moreover, an increased in IL-10 responses of PBMCs after treatment is higher than the levels during the pre-treatment period (53). In addition, the intracellular IFN-gamma responses of PBMCs to *S. sanguinis* is suppressed (54). These results are due to the immunomodulatory effects of the azithromycin (53, 54). Interestingly, IL-10 is identified as a BD susceptibility loci in a genome wide association study (90, 91) whose results give clues for the polarisation of anti-inflammatory cytokine-response (91).

Apremilast as a phosphodiesterase 4 blocker controls oral ulcer activity and decreases oral ulcer-related pain in BD by modulating inflammatory pathways. The elimination of oral ulcers starts within the first week of the treatment (92, 93).

Since poor oral health with ulceration and infective foci are implicated in pathogenesis of the BD, regular dental check-up are important. From the patients' perspective, the planning of dental visits could be affected by disease activity, oral ulcer related factors or using immunosuppressive treatments. Dental treatment should not be undertaken in BD patients who have active disease, as they will experience an exacerbation of their BD symptoms which may also be activated by dental treatment secondary to mechanical trauma causing a break in the epithelium exposing circulation to the oral environment (32). In addition, lack of infection control could be seen immunocompromised patients (27). The motivation of oral hygiene is fairly high in female BD patients with active oral ulcers (94). Moreover, individual factors (psychosocial factors, education, income, gender) and National Oral Health policies are all associated with an individual's oral health (95) (Figure 1).

## Promotion of Oral Health as an Etiologic Factor in Behçet's Disease

As Oral Health policies and systems to deliver oral health care varies widely between different among countries (82), the delivery of care may be reliant on an “out of pocket payment system” with minimal income to cover costs incurred. Clues to these background factors may be found in careful questioning. Questions about reason for the last dental visit (control vs. emergency care), infrequency of dental visit, lack of regular oral hygiene habits and presence of pain and/or discomfort provide important indicators for physicians about oral health. Oral health education should be part of the BD clinic visit. Patients should be motivated to engagement good hygiene practises, attend regular dental check-ups to shift from treatment-oriented to preventive-oriented pattern. The recall periods should be varied according to individual risk factors (28, 32, 33, 46, 47, 83) (Figure 1).

Finally, from the Oral health perspective, a series of complex interactions are observed in the pathogenesis of BD. Since oral microorganisms have an important role in the onset of the activation of the disease, the elimination of infective foci and the protection of oral health are the fundamental of the disease management. It is crucial to have close working relationship between oral and general physicians for the promotion of both oral and subsequently general health. Achieving good oral health will greatly contribute to the improved prognosis in BD.

## Author Contributions

All authors listed have made a substantial, direct and intellectual contribution to the work, and approved it for publication.

## Conflict of Interest

The authors declare that the research was conducted in the absence of any commercial or financial relationships that could be construed as a potential conflict of interest.
